# Correlation of *HLA-DQ* and *TNF-α* gene polymorphisms with ocular myasthenia gravis combined with thyroid-associated ophthalmopathy

**DOI:** 10.1042/BSR20160440

**Published:** 2017-03-27

**Authors:** Hong-Wei Yang, Ying-Xue Wang, Jie Bao, Shu-Hui Wang, Ping Lei, Zhao-Lin Sun

**Affiliations:** 1 Geriatric Ward of Neurology, Tianjin Geriatrics Institute, Tianjin Medical University General Hospital, Tianjin 300052, P.R. China; 2 Department of Endocrinology and Metabolism, The First Affiliated Hospital of Jinan University, Shipai, Guangzhou 510630, P.R. China; 3 Department of Rehabilitation Medicine, Tianjin Medical University General Hospital, Tianjin 300052, P.R. China; 4 Department of Neurology, Beijing Friendship Hospital, Capital Medical University, Beijing 100050, P.R. China; 5 Department of Neurology, Affiliated Hospital of Medical College, Qingdao University, Qingdao 266000, P.R. China

**Keywords:** Antibody, Gene polymorphism, HLA-DQ, Ocular myasthenia gravis, TNF-α, Thyroid associated ophthalmopathy

## Abstract

The present study aims to explore the correlation of human leucocyte antigen (*HLA*)*-DQ* and tumour necrosis factor (*TNF*)*-α* gene polymorphisms with ocular myasthenia gravis (OMG) combined with thyroid-associated ophthalmopathy (TAO). From March 2009 to March 2015, 56 OMG patients complicated with TAO (OMG + TAO group), 134 patients diagnosed with OMG only (OMG group) and 236 healthy individuals (control group) were enrolled in the present study. PCR-sequence specific primer (PCR-SSP) was used for *HLA-DQ* genotyping and PCR-restriction fragment length polymorphism (PCR-RFLP) for *TNF-α* genotyping. ELISA kit was applied to detect acetylcholine receptor antibody (AchRAb) level and chemiluminescence immunoassay (CLIA) to measure thyroid-associated antibody (T-Ab) level. Logistic regression analysis was carried out to analyse the risk factors for OMG combined with TAO. DQA1*0103 showed lower frequency in the OMG group than in the control group. DQA1*0301 showed increased and DQB1*0601 showed decreased frequency in the OMG + TAO group. DQB1*0501 showed higher frequency in the OMG and OMG + TAO groups than in the control group. Patients carrying *TNF-α* -863C > A (CA + AA) might confront with greater risks of OMG combined with TAO. Frequency of DQA1*0103/*0301 and DQB1*0501/*0601, and *TNF-α* -863C > A, -238G > A and -308G > A were associated with the levels of AchRAb and T-Ab. *TNF-α* -863C > A (CA + AA) and high level of T-Ab were risk factors for OMG combined with TAO. Our results demonstrate that *TNF-α* -863 polymorphism is possibly correlated with the risk of OMG combined with TAO.

## Introduction

Myasthenia gravis (MG) is an autoimmune disease resulting from an immunological response against the acetylcholine receptor (AChR) at the neuromuscular junction, leaving the voluntary muscles weak [1]. With an overall prevalence of 12 cases per 100000, ocular MG (OMG) shares the very same process with MG and affects almost half of MG patients, but its symptoms are restricted to the extraocular and levator palpebrae muscles, which take the form of diplopia and ptosis [2]. OMG can lead to visual disabilities and disrupt daily activities, and approximately 44% OMG patients are reported to develop generalized MG, which even poses a threat to survival [3]. Therefore, early diagnosis and treatment are fundamentally important. Graves’ disease (GD) is a common autoimmune thyroid disorder and thyroid-associated ophthalmopathy (TAO) can affect 30–50% of GD patients and is characterized as orbital inflammation and expansion of fat and extraocular muscles [4]. The concurrence of GD and MG, though with relatively rare incidences, is particularly popular in the Asian women with age under 50 years [5,6]. Currently, there is no standard method for the treatment of OMG combined with TAO and the use of thymectomy and rituximab is quite controversial [7,8]. Anti-AChR antibodies are found to impair neuromuscular transmission and induce exacerbating receptor condition, causing increasingly reduced AChR availability and ensuing OMG [9]. Therefore, a new way to deal with OMG is very significant on genetic molecular basis.

Human leucocyte antigen (*HLA*) genes are vital in modulating the immune response to viral pathogens [10]. HLA class II, especially highly polymorphic HLA-DR and -DQ alleles, is crucial to human immune responses due to their functions in stemming the predisposition or protecting human bodies from certain diseases [11]. Previous studies have reported that *HLA-DQ* gene polymorphisms are closely associated with the development of MG [12,13]. Tumour necrosis factor (TNF)-α is a prototypical and pro-inflammatory cytokine, which can signal cell survival, proliferation and activation or even death via its type 1 receptor (TNFR1) [14]. Genetic polymorphisms in the promoter region of TNF-α are found to be involved in regulating levels and related to a large number of inflammatory and malignant diseases such as lung cancer and TAO [15,16]. However, the specific mechanisms behind these molecules still remain elusive. ‘See-saw’ relationship has been discussed very early between MG and hyperthyroidism [17,18]. While a previous study demonstrates a reverse ‘see-saw’ relationship between MG and GD on the basis of their clinical and immunological features and also suggests that HLA-DQ3 may play a pathogenic role in the concomitance of the two diseases [19]. Although accumulating support has been given to the reverse one, scholars at home and abroad have not reached a unified understanding of the mechanism of the concurrence of OMG and GD, which may be involved in various aspects of immunity and abnormal genes. And as the *HLA-DQ* gene has been shown as a genetic marker for resistance to autoimmune thyroid diseases [20] and TNF-α receptor blockers are involved in MG [21], we hypothesized that *HLA-DQ* and *TNF-α* gene polymorphisms may be a promising genetic target of OMG combined with TAO. Consequently, the present study is carried out to explore the correlation of *HLA-DQ* and *TNF-α* gene polymorphisms with OMG combined with TAO and related antibodies, with hope to lay molecular basis for early diagnosis of the disease.

## Materials and methods

### Ethics statement

The present study was approved by the Ethics Committee of Tianjin Geriatrics Institute, Tianjin Medical University General Hospital and all participants signed an informed consent.

### Study subjects

From March 2009 to March 2015, 190 patients (82 males and 108 females, mean age: 33.59 ± 7.84 years) with OMG receiving treatment in Tianjin Geriatrics Institute, Tianjin Medical University General Hospital were selected in the present study, including 56 patients complicated with TAO (the OMG + TAO group) and 134 patients diagnosed with OMG only (the OMG group). Inclusion criteria for OMG patients: (i) patients who were consistent with Chinese Medical Syndrome Differentiation Standards; (ii) patients who were in conformity to Western Diagnosis of OMG [22]; (iii) patients who were diagnosed as extraocular muscle involvement only with no other muscles involvement, no electrophysiology and no evidence of progression to other muscles. Exclusion criteria for OMG patients: (i) patients with affected muscles in parts other than in ocular part; (ii) patients diagnosed with other severe systemic diseases; (iii) patients with severe skin disease, mental disorder or other diseases involving heart, brain, liver and kidney; (iv) women under pregnancy and lactation and those who can seek self-relief out of psychological and mental concerns. Inclusion criteria for OMG patients combined with TAO: (i) patients who were consistent with inclusion criteria for OMG patients; (ii) patients who were in conformity to criteria for Western Diagnosis of TAO [23]. Exclusion criteria for OMG patients combined with TAO: (i) patients who were consistent with exclusion criteria for OMG patients; (ii) patients with thyroid crisis; (iii) patients with severe mental disorder; (iv) patients who were diagnosed with other severe systemic diseases. Concurrently, 236 healthy individuals (94 males and 142 females, mean age: 32.61 ± 8.23 years) who had no relationship with the included OMG patients and who received physical examinations in Tianjin Geriatrics Institute, Tianjin Medical University General Hospital were selected as the control group. There was no obvious difference among the three groups in the baseline data (*P*>0.05).

### *HLA-DQ* genotyping

PCR-sequence specific primer (PCR-SSP) was employed to detect *HLA-DQ* gene polymorphism. Peripheral venous blood (10 ml) was collected from all the subjects, 5 ml of which was placed in an EDTA anticoagulation test tube, the rest of which was placed in common tubes for antibody detection. Whole blood genomic DNA kit (Promega Corp., Madison, WI, U.S.A.) was used to extract whole blood genomic DNA. Sequence specific primers (SSP) of DQA1 and B1 were designed consulting Bunce et al. [24] and internal reference primers (IRP) (forward (F): 5′-TGC CAA GTG GAGCACCCAA-3′, reverse (R): 5′-GCATCTTGCTCTGTGCAGAT, 796 bp) were designed according to Olerup et al. [25], which were both synthesized by Shanghai Sangon Biological Engineering Technology & Services Co., Ltd. (Shanghai, China). PCR-SSP classification of DQA1 and DQB1 was processed in accordance with Olerup et al. [25]. Reaction system for PCR was 20 μl, including DNA 2 μl, Taq enzyme 1 μl, mixture of primers (SSP + IRP + 56 mM KCl + 1.7 mM MgCl_2_) 8 μl, dNTP 1.6 μl and addition of ddH_2_O until 20 μl altogether. Reaction conditions for PCR were: pre-denaturation at 94°C for 4 min, denaturation at 94°C for 20 s, annealing at 65°C for 50 s, extension at 72°C for 20 s, total 30 cycles, ending with extension at 72°C for 10 min. After that, 0.5%× TBE solution was used to prepare 2% agarose gel, where 1 μg/ml of Ethidium Bromide (EB) was added. Then the mixture received 30-min electrophoresis under gel condition with a voltage of 5 V/cm. UV imaging system was employed to observe which SSP caused specificity amplified bands so as to conduct DQA1 and DQB1 genotyping.

### *TNF-α* genotyping

PCR-restriction fragment length polymorphism (PCR-RFLP) was used to detect *TNF-α* gene polymorphism and Primer Premier 5.0 software was employed to design PCR primers, which were synthesized by Shanghai Sangon Biological Engineering Technology & Services Co., Ltd. (Shanghai, China) (Table 1), of which, TNF-863 site entailed mismatched base PCR, namely, a mismatched base (A→C) was introduced at the second site of 3′ tail of downstream primers so as to mimic artificially a taiI enzyme restriction site. Reaction system for PCR was 20 μl: 2 μl of DNA genome, 1 μl of primers of downstream and upstream each, 2.5 μl of 10 buffer, 1.5 μl of MgCl_2_ (25 mM), 2 μl of dNTP, 0.2 μl of Taq enzyme (TIANGEN Biotechnology Co. Ltd., Beijing, China) and 9.8 μl of ddH_2_O. Reaction conditions for PCR were: 30 cycles of pre-denaturation at 94°C for 2 min, denaturation at 94°C for 30 s, annealing at 55°C for 45 s and extension at 72°C for 45 s, ending with 10-min extension at 72°C. PCR products received enzyme digestion at 37°C overnight and were identified by 3% agarose gel electrophoresis to locate *TNF-α* promoter region, including -238G > A, -308G > A, -857C > T, -863C > A and -1031T > C genotypes.

**Table 1:** Primer sequences of *TNF-α* for PCR-RFLP

Locus	Primer sequence	Products (bp)
-238G **>** A	F: 5′-AGAAGACCCCCCTGGGAACC-3′	152
	R: 5′-ATCTGGAGGAAGCGGTAGTG-3′	
-308G **>** A	F: 5′-AGGCAATAGGTTTTGAGGGCCAT-3′	107
	R: 5′-TCCTCCCTGCTCCGATTCCG-3′	
-857C **>** T	F: 5′-AAGTCGAGTATGGGGACCCCCCGTTAA-3′	131
	R: 5′-CCCCAGTGTGTGGCCATATCTTCTT-3′	
-863C **>** A	F: 5′-GGCTCTGAGGAATGGGTTAC-3′	125
	R: 5′-CTACATGGCCCTGTCTTCGTTACG-3′	
-1031T **>** C	F: 5′-ATGTGATGGACTCACCAGGT-3′	264
	R: 5′-CTCTACATGGCCCTGTCTT-3′	

### Enzyme-linked immunosorbent assay (ELISA)

Peripheral blood without anticoagulation (4 ml) was collected and placed at room temperature for 30 min, followed by centrifugation (3000 rev/min) for 15 min to obtain the serum. ELISA kit (R S R Ltd., Cardiff, U.K.) was used to detect the absorbance (*A*) value of AChR antibody (AChRAb) (450 nm) and thus its concentrations were calculated. According to the standard for positive AChRAb, standard curve is drawn in conformity with *A* value of standards and corresponding concentrations are found in line with *A* value of samples. When the concentration reached over 0.2 nmol/l, the antibody was positively expressed [26]. Chemiluminescence immunoassay (CLIA) was employed to assess the serum levels of thyroglobulin antibody (TgAb), thyrotrophin receptor antibody (TRAb) and thyroid peroxidase antibody (TPOAb). Normal reference range: TgAb, 0–115 IU/ml; TRAb, 0–15 IU/ml; TPOAb, 0–34 IU/ml; over the upper limit proved to be positive [27]. If one of the three antibodies was positive, expression of thyroid-associated antibody (T-Ab) was considered as positive.

### Statistical analysis

Statistical software of SPSS 20.0 was employed for statistical analysis, with measurement data presented as mean ± S.D. The Student’s *t* test was employed for the comparison among groups, whereas ANOVA was used for comparisons among the three groups. Enumeration data were presented as a percentage or ratio and examined with Chi-square. Relative risks of genotype were presented as odds ratio (OR) or 95% confidence interval (95% CI). Group representation of the samples was assessed using the Hardy–Weinberg equilibrium test. Logistic regression analysis was conducted for risk factors of OMG patients combined with TAO. *P* value presented two-tailed probability and *P*<0.05 was considered as statistically significant.

## Results

### Correlation of *HLA-DQ* gene polymorphism with OMG and OMG combined with TAO

As shown in Table 2, compared with the control group, the OMG group presented notably decreased frequency of DQA1*0103 allele (OR =0.034, 95% CI =0.057–0.958; *P*<0.05), and the OMG + TAO group exhibited significantly increased frequency of DQA1*0301 allele (OR =9.116, 95% CI =0.498–167.100; *P*<0.05). Compared with the control group, DQB1*0501 frequency was remarkably increased in the OMG and OMG + TAO groups, indicating that 0501 allele might increase the risk of OMG and TAO-combined OMG (OR =3.388, 95% CI =0.979–11.730; OR =5.029, 95% CI =0.950–26.620; both *P*<0.05). DQB1*0601 frequency was significantly reduced in the OMG + TAO group (OR =0.110, 95% CI =0.009–1.347; *P*<0.05).

**Table 2:** Phenotypic count of *HLA-DQ* gene polymorphism in the control, OMG + TAO and OMG groups

Locus	Allele	Control	OMG + TAO	OMG	P*	OR (95% CI)*	*P*^†^	OR (95% CI)^†^	*P*^‡^	OR (95% CI)^‡^
DQA1	0101	8	0	5						
	0102	32	12	34	0.092	6.538 (0.350–122.000)	0.390	1.700 (0.503–5.744)	0.192	0.251 (0.013–4.877)
	0103	41	4	6	0.381	1.843 (0.090–37.560)	0.034	0.234 (0.057–0.958)	0.099	0.131 (0.006–3.020)
	0104	25	5	12	0.215	3.667 (0.183–73.500)	0.693	0.768 (0.207–2.854)	0.168	0.207 (0.010–4.427)
	0201	36	8	19	0.190	3.959 (0.207–75.560)	0.791	0.844 (0.242–2.942)	0.160	0.209 (0.010–4.213)
	0301	34	18	22	0.047	9.116 (0.498–167.100)	0.956	1.035 (0.300–3.576)	0.053	0.111 (0.006–2.135)
	0302	2	0	0	-	-	0.283	0.309 (0.012–7.744)	-	-
	0401	8	1	2	0.331	3.000 (0.106–84.630)	0.340	0.400 (0.059–2.703)	0.168	0.152 (0.004–5.188)
	0501	34	7	18	0.207	3.696 (0.192–71.330)	0.795	0.847 (0.241–2.972)	0.177	0.224 (0.011–4.584)
	0601	16	1	16	0.484	1.545 (0.057–42.180)	0.482	1.600 (0.430–5.960)	0.579	1.000 (0.035–28.330)
DQB1	0201	9	2	5						
	0301	26	6	17	0.967	1.038 (0.177–6.104)	0.799	1.177 (0.336–4.120)	0.896	1.133 (0.172–7.472)
	0302	12	3	10	0.908	1.125 (0.154–8.209)	0.563	1.500 (0.378–5.954)	0.787	1.333 (0.165–10.750)
	0303	17	3	10	0.818	0.794 (0.112–5.658)	0.934	1.059 (0.276–4.060)	0.271	1.333 (0.165–10.750)
	0401	3	0	0	0.425	0.543 (0.021–14.360)	0.218	0.247 (0.011–5.727)	-	-
	0501	17	19	32	0.043	5.029 (0.95026.620)	0.047	3.388 (0.979–11.730)	0.654	0.674 (0.119–3.822)
	0504	11	2	6	0.855	0.818 (0.095–7.020)	0.981	0.982 (0.224–4.306)	0.876	1.200 (0.121–11.870)
	0601	41	1	6	0.044	0.110 (0.009–1.347)	0.050	0.264 (0.0661.057)	0.515	2.400 (0.165–34.950)
	0602	46	8	20	0.778	0.783 (0.142–4.313)	0.692	0.783 (0.233–2.632)	1.000	1.000 (0.160–6.258)
	0603	16	4	6	0.902	1.125 (0.171–7.403)	0.592	0.675 (0.160–2.852)	0.628	0.600 (0.076–4.763)
	0604	22	5	13	0.987	1.023 (0.167–6.277)	0.925	1.064 (0.293–3.867)	0.968	1.040 (0.1507.220)
	0606	16	3	9	0.866	0.844 (0.118–6.034)	0.986	1.013 (2.589–3.964)	0.865	1.200 (0.147–9.773)

*, comparison between the control and OMG + TAO groups; ^†^, comparison between the control and OMG groups; ^‡^, comparison between the OMG + TAO and OMG groups; Control, the healthy individuals.

### Correlation of *TNF-α* gene polymorphism with OMG and OMG combined with TAO

Goodness of Fit was employed for Hardy–Weinberg equilibrium test on the locus in the promoter region of *TNF-α* in the control, OMG and OMG + TAO groups, namely, -238G > A, -308G > A, -1031C > T, -857C > T and -863C > A. The results showed that among the three groups, the actual values of the five genotypes of *HLA-DQ* and their allele distributions were equal to the expected values, which conformed to Hardy–Weinberg equilibrium, suggesting that the samples were representative.

At the locus -863C > A in the *TNF-α* promoter region, the OMG + TAO group showed notably higher frequencies of CA + AA genotype and A allele than the control and OMG groups (all *P*<0.05). No significant difference was found in the frequency of -863C > A genotype between the control and OMG groups (*P*>0.05). In addition, there were no significant differences in the frequency of the genotypes and alleles of -238G > A, -308G > A, -857C > T and -1031T > C in the *TNF-α* promoter regions among the control, OMG and OMG + TAO groups (all *P*>0.05) (Table 3).

**Table 3:** Frequency distribution of *TNF-α* gene polymorphism in the control, OMG + TAO and OMG groups

Locus	Allele	Control	OMG + TAO	OMG	P*	OR (95% CI)*	P^†^	OR (95% CI)^†^	*P*^‡^	OR (95% CI)^‡^
	GG	214	50	114			Ref.			
	GA	22	6	19	0.750	0.857 (0.330–2.224)	0.145	0.617 (0.321–1.187)	0.508	1.389 (0.523–3.687)
-238G/A	AA	0	0	1	-	-	0.172	0.178 (0.007–4.407)	0.508	1.323 (0.053–33.070)
	GA + AA	22	6	20	0.750	0.857 (0.330–2.224)	0.103	0.586 (0.307–1.119)	0.441	1.462 (0.554–3.861)
	G	450	106	247			Ref.			
	A	22	6	21	0.757	0.864 (0.3422.183)	0.076	0.575 (0.310–1.067)	0.391	1.502 (0.589–3.828)
	GG	212	46	113			Ref.			
	GA	24	10	20	0.107	0.521 (0.233–1.163)	0.166	0.640 (0.339–1.208)	0.628	0.814 (0.354–1.873)
-308G/A	AA	0	0	1	-	-	0.172	0.178 (0.007–4.409)	0.524	1.229 (0.049–30.750)
	GA + AA	24	10	21	0.107	0.521 (0.233–1.163)	0.120	0.609 (0.325–1.142)	0.710	0.855 (0.374–1.956)
	G	448	102	246			Ref.			
	A	24	10	22	0.118	0.546 (0.253–1.178)	0.091	0.599 (0.329–1.091)	0.818	0.912 (0.417–1.995)
	CC	170	46	96			Ref.			
	CT	63	10	35	0.156	1.705 (0.811–3.582)	0.947	1.016 (0.627–1.648)	0.194	1.677 (0.764–3.680)
-857C/T	TT	3	0	3	0.369	1.909 (0.097–37.650)	0.484	0.565 (0.112–2.854)	0.233	3.373 (0.171–66.710)
	CT + TT	66	10	38	0.121	1.786 (0.851–3.746)	0.936	0.981 (0.612–1. 571)	0.129	1.821 (0.834–3.974)
	C	403	102	227			Ref.			
	T	69	10	41	0.114	1.746 (0.869–3.510)	0.803	0.948 (0.623–1.442)	0.097	1.842 (0.888–3.822)
	CC	183	30	95			Ref.			
	CA	53	21	37	0.006	2.417 (1.279–4.566)	0.233	0.744 (0.457–1.211)	0.087	1.797 (0.915–3.530)
-863C/A	AA	0	5	2	<0.001	66.180 (3.566–1228)	0.051	0.104 (0.005–2.192)	0.006	7.917 (1.460–42.940)
	CA + AA	53	26	39	<0.001	2.992 (1.629–5.495)	0.155	0.706 (0.436–1.143)	0.022	2.111 (1.109–4.020)
	C	419	81	227						
	A	53	31	41	<0.001	3.026 (1.829–5.004)	0.110	0.700 (0.452–1.086)	0.005	2.119 (1.246–3.604)
	CC	157	42	92			Ref.			
	CT	75	14	38	0.287	1.433 (0.737–2.786)	0.542	1.157 (0.725–1.846)	0.555	1.239 (0.607–2.529)
-1031T/C	TT	4	0	4	0.302	2.429 (0.128–46.030)	0.453	0.586 (0.143–2.400)	0.179	4.135 (0.218–78.610)
	CT + TT	79	14	42	0.221	1.510 (0.778–2.929)	0.675	1.102 (0.700–1.736)	0.382	1.370 (0.676–2.776)
	C	389	98	222						
	T	83	14	46	0.885	1.030 (0.693–1.530)	0.885	1.030 (0.693–1.530)	0.256	1.450 (0.762–2.762)

*, comparison between the control and OMG + TAO groups; ^†^, comparison between the control and OMG groups; ^‡^, comparison between the OMG + TAO and OMG groups; Ref., reference; Control, the healthy individuals.

### Relationship between *HLA-DQ* and *TNF-α* gene polymorphisms and AChRAb

The positive rate of AChRAb in patients with *HLA-DQ* DQA1*0301 and DQB1*0501 was notably higher in the OMG + TAO group than in the OMG group (both *P*<0.05); and the positive rate of AChRAb in patients with DQA1*0103 and DQB1*0601 was remarkably lower in the OMG + TAO group than in the OMG group (both *P*<0.05) (Table 4). The positive rate of AChRAb in the patients carrying CA + AA of -863C > A in the *TNF-α* promoter region was notably higher in the OMG + TAO group than those in the OMG group (*P*<0.05) (Table 5).

**Table 4:** Relationship between *HLA-DQ* gene polymorphism and AChRAb

Locus	Allele	OMG + TAO		OMG		*P*
		(+)	(–)	(+)	(–)	
	0101	0	0	2	3	-
	0102	5	7	16	18	0.747
	0103	1	3	6	0	0.011
	0104	3	2	3	9	0.169
DQA1	0201	2	6	2	17	0.334
	0301	10	8	5	17	0.033
	0302	0	0	0	0	-
	0401	1	0	1	1	0.387
	0501	2	5	2	16	0.285
	0601	0	1	4	12	0.568
	0201	1	1	2	3	0.809
	0301	3	3	8	9	0.901
	0302	1	2	4	6	0.835
	0303	2	1	5	5	0.612
DQB1	0401	0	0	0	0	-
	0501	12	7	5	27	0.001
	0504	1	1	2	4	0.673
	0601	0	1	6	0	0.008
	0602	2	6	4	16	0.771
	0603	0	4	1	5	0.389
	0604	1	4	2	11	0.814
	0606	1	2	2	7	0.700

+, positive; –, negative.

**Table 5:** Relationship between *TNF-α* gene polymorphism and AChRAb

Genotype	OMG + TAO		OMG		*P*
	(+)	(–)	(+)	(–)	
-238G > A					0.297
GG	22	28	38	76	
GA + AA	2	4	3	17	
-308G > A					0.327
GG	22	24	36	77	
GA + AA	2	8	5	16	
-857C > T					0.237
CC	21	25	32	64	
CT + TT	3	7	9	29	
-863C > A					<0.001
CC	15	15	37	58	
CA + AA	9	17	4	35	
-1031T > C					0.051
CC	22	20	36	56	
CT + TT	2	12	5	37	

+, positive; –, negative.

### Relationship between *HLA-DQ* and *TNF-α* gene polymorphisms and T-Ab

There was significant difference in AchRAb and T-Ab among the three groups. The OMG + TAO group exhibited higher positive rates of AchRAb and T-Ab than the OMG and the control groups (all *P*<0.05). The positive rate of T-Ab in patients with *HLA-DQ* DQA1*0301 and DQB1*0501 was remarkably higher in the OMG + TAO group than in the OMG group (both *P*<0.05). However, the positive rate of T-Ab in patients with DQA1*0301 and DQB1*0601 was remarkably lower in the OMG + TAO group than in the OMG group (both *P*<0.05) (Table 6). The positive rate of T-Ab in patients carrying CA + AA of -863C > A in the *TNF-α* promoter region was notably higher in the OMG + TAO group than the patients in the OMG group (*P<*0.05); the positive rate of T-Ab in patients carrying CA + AA of -238G > A and -308G > A were notably higher in the OMG + TAO group than in the OMG group (both *P<*0.05) (Table 7).

**Table 6:** Relationship between *HLA-DQ* gene polymorphism and T-Ab

Locus	Allele	OMG + TAO		OMG		*P*
		(+)	(–)	(+)	(–)	
	0101	0	0	1	4	-
	0102	6	6	14	20	0.596
	0103	1	3	6	0	0.011
	0104	4	1	5	7	0.149
DQA1	0201	2	6	2	17	0.334
	0301	12	6	5	17	0.005
	0302	0	0	0	0	-
	0401	1	0	1	1	0.387
	0501	2	5	2	16	0.285
	0601	0	1	4	12	0.568
	0201	2	0	2	3	0.147
	0301	3	3	7	10	0.708
	0302	1	2	4	6	0.835
	0303	2	1	4	6	0.416
DQB1	0401	0	0	0	0	-
	0501	13	6	7	25	0.001
	0504	1	1	1	5	0.346
	0601	0	1	6	0	0.008
	0602	3	5	4	16	0.334
	0603	1	3	1	5	0.747
	0604	1	4	2	11	0.814
	0606	1	2	2	7	0.700

+, positive; –, negative.

**Table 7:** Relationship between *TNF-α* gene polymorphism and T-Ab

Locus	Allele	OMG + TAO		OMG	*P*
		(+)	(–)	(+)	
-238G > A					0.034
GG	26	24	39	75	
GA + AA	2	4	1	19	
-308G > A					0.017
GG	24	22	36	77	
GA + AA	4	6	4	17	
-857C > T					0.057
CC	24	22	34	62	
CT + TT	4	6	6	32	
-863C > A					<0.001
CC	16	14	38	57	
CA + AA	12	14	2	37	
-1031T > C					0.053
CC	23	19	34	58	
CT + TT	5	6	6	36	

+, positive; –, negative.

### Logistic regression analysis for risk factors of OMG patients combined with TAO

Logistic regression analysis was carried out with whether OMG is complicated with TAO or not as a dependent variable, and the locus -863C > A, -238G > A and -308G > A in the *TNF-α* promoter region and DQA1*0103 and *0301, DQB1*0501 and *0601 and expressions of AChRAb and T-Ab as independent variables. The results revealed that *TNF-α* -863C > A (CA + AA) genotype and high expression of T-Ab were risk factors for OMG patients combined with TAO (Table 8).

**Table 8:** Logistic regression analysis for risk factors of OMG patients combined with TAO

Variable	*B*	S.E.M.	Wald	df	Sig.	Exp (B)	95% CI
AchRAb	–0.295	0.671	0.193	1	0.661	0.745	0.200–2.775
T-Ab	1.325	0.665	3.966	1	0.046	3.762	1.021–13.863
DQA1*0103	0.769	0.786	0.958	1	0.328	2.158	0.462–10.65
DQA1*0301	0.756	0.401	3.561	1	0.059	2.131	0.971–4.674
DQB1*0501	0.780	0.423	3.401	1	0.065	2.180	0.952–4.993
DQB1*0601	–0.977	1.222	0.638	1	0.424	0.377	0.034–4.133
*TNF-α* -863C > A	1.276	0.418	9.304	1	0.002	3.584	4.578–8.139
*TNF-*α -238G > A	–0.379	0.558	0.463	1	0.496	0.684	0.229–2.042
*TNF-α* -308G > A	0.120	0.481	0.062	1	0.803	1.127	0.439–2.892

*B*, partial regression coefficient; df, degree of freedom; Exp (B), adjusted OR; Sig., significant *P* value; Wald, Wald χ^2^.

## Discussion

Reported as one of the main reasons for discomfort and decreased quality of life, OMG combined with TAO requires immediate attention [28]. However, treatments for OMG and TAO do not seem to have any achievements substantially during the past few decades [2,4]. Nonetheless, previously, genetic polymorphism has been reported to be associated with MG, giving us inspiration to look at it from genetic perspectives [12].

It was revealed in the study that *HLA-DQ* DQB1*0501 and *TNF-α* -863C > A showed higher frequencies in the OMG and OMG + TAO groups than in the control group, suggesting that gene polymorphisms of *HLA-DQ* and *TNF-α* probably increased the risks of OMG combined with TAO. Many factors are found associated with MG, including MHC (whose epithet in human beings is referred to as HLA), AchR, Ig, T-cell antigen receptor (TCR) and interleukin (IL)-1 genes [29]. HLA II molecules are responsible for presenting pathogenic epitopes to CD4+ T cells, which are important in the manifestation and perpetuation of MG by producing high-affinitive Abs [13]. How CD4+ T cells are selected in the thymus is regulated by HLA class II molecules, whose polymorphism at this locus is very likely to give rise to susceptibility of OMG coexisting with TAO. Many previous studies have demonstrated the association between MG and *HLA-DQ* polymorphism [12,30], and patients with thyroid disease are prone to suffer from MG [6]. As TAO and OMG are related to thyroid, we assume that there must be similar pathogenesis between these two diseases. Therefore, *HLA-DQ* polymorphism may be involved in both TAO and OMG, which however, could be supported by few previous studies and further studies are still needed to confirm the result. *TNF-α* genes are situated in the region of HLA class III (250-kb centromeric of the HLA-B and 850-kb telomeric of the class II *HLA-DR* genes in humans) and were found to be involved in the incidence and progression of some contagious and autoimmune diseases [31]. TNF-α enhanced the expression of HLA class I and adhesion molecules on thyrocytes, and increased expression of the molecules on thyrocytes would promote the autoimmune responses related to the pathogenesis of TAO, besides the polymorphic allele of *TNF-α* gene at position 308 (G to A, -308A) or 238 (G to A, -238A) was found to be involved in severity of and susceptibility to some autoimmune and infectious diseases [32]. Skeie et al. [33] have also found that presence of *TNF-α* alleles correlates with the onset of MG [33]. Additionally, Yan et al. [16] have demonstrated that allele A of *TNF-α* at position -863 is probably linked to TAO, in male patients in particular, which is completely consistent with our findings.

We also found that the frequency distributions of DQA1*0301, DQB1*0501 and *TNF-α* -863C > A (CA + AA), -238G > A (GG) and -308G > A (GG) genotypes are associated with the positive rates of AchRAb and T-Ab in the OMG + TAO group. Furthermore, the logistic regression analysis confirmed that the CA + AA genotype of *TNF-α* -863C > A and high expression of T-Ab are risk factors for OMG combined with TAO. *TNF-α* -863C > A is associated with TNF-α secretion [34], and TNF-α may directly destroy AChR or promote the differentiation and growth of B cells resulting in an enhanced production of AChRAb [35], stimulating high expressions of AchRAb. Therefore, they are proceeding to cause increasingly reduced AChR availability and ensuing OMG by impairing neuromuscular transmission and inducing exacerbating receptor condition [9]. Additionally, it has long been recognized that positive AchRAb is synonymous with the onset of OMG [36]. Furthermore, as to *HLA-DQ* polymorphism, a result consistent with ours has also been reported by Jonsdottir et al. [37] that *HLA-DQ* is associated with positive T-Ab, further supporting our findings. However, despite the discovery of correlation between *HLA-DQ* and positive T-Ab in patients with TAO-combined OMG, we failed to figure out the mechanism behind it, which awaits further studies to elucidate it.

To summarize, we can come to the conclusion that TNF-α -863 polymorphism is likely to be associated with OMG combined with TAO, promising to lay a molecular basis for early diagnosis of the disease. However, the present study also has its own limitations. Due to the limited sample size and *DQA1* and *DQB1* gene polymorphism, which have multiple alleles, the present study showed significant difference in the AChRAb between the TAO and OMG groups with no obvious difference in distribution of *HLA-DQ* alleles between the OMG + TAO group and the OMG group, which may be further confirmed in the future and may be a research direction. And more similar studies with larger sample size are needed to be launched with prudence so as to verify our results and further elucidate possible mechanisms.

## Abbreviations

Abs: AChr antibodies
AChR: acetylcholine receptor
AChRAb: AChR antibody
ddH20: double distilled water
F: forward
GD: Graves’ disease
HLA: human leucocyte antigen
IRP: internal reference primers
MG: myasthenia gravis
OMG: ocular MG
OR: odds ratio
PCR-RFLP: PCR-restriction fragment length polymorphism
PCR-SSP: PCR-sequence specific primer
R: reverse
T-Ab: thyroid-associated antibody
TAO: thyroid-associated ophthalmopathy
TBE: tris-borate-EDTA
TgAb: thyroglobulin antibody
TNF: tumour necrosis factor
TRAb: thyrotrophin receptor antibody
TPOAb: thyroid peroxidase antibody
95% CI: 95% confidence interval
